# Acute exogenous intoxications in childhood: factors related to hospitalization

**DOI:** 10.1590/1984-0462/2024/42/2023028

**Published:** 2023-12-11

**Authors:** Juliana Gabriela Burgardt Werner, Vanessa Borges Platt

**Affiliations:** a Hospital Infantil Joana de Gusmão – Florianópolis, SC, Brazil.; b Universidade Federal de Santa Catarina – Florianópolis, SC, Brazil.

**Keywords:** Poisoning, Accidental injuries, Child, Emergency service, Intoxicação, Lesões acidentais, Criança, Serviço Hospitalar de Emergência

## Abstract

**Objective:**

To describe the profile of children assisted in the Emergency Room of a Children reference Hospital in the South of Brazil, victims of acute poisoning between 2016 and 2021, to characterize the toxic agents and to present the factors related to hospitalization.

**Methods:**

Retrospective, descriptive and observational study with data collection from medical records at a children’s hospital from July 2016 to June 2021 based on the compulsory notification forms. The characteristics of victims, of the incident, the type and class of the substance involved, the procedures demanded and the need for the Intensive Care Unit were evaluated. The outcome considered was hospitalization. Absolute and relative frequencies were calculated for the categorical variables and measures of central tendency and dispersion for the numerical ones. Binary logistic regression was performed to identify variables related to hospitalization.

**Results:**

There were 411 consultations, with the mean age of 7.2±5.5 years, and predominance of females (59.9%). Most of the poisoning occurred at home (82.1%) and orally (93.7%). Chemicals or cleaning products were the main agents in children up to 1 year of age, whereas in the other age groups accidents occurred most frequently with medicines. Hospital admission occurred in 38.7% of the cases, with related variables being: number of agents, type of substance involved, medication that acts on the Central Nervous System, recurrence, motivation (accidental/intentional), and performance of simple exams.

**Conclusions:**

More preventive actions are needed, such as legislation, as well as greater guidance to parents on how to store products in the domestic environment, in order to reduce the number of exogenous intoxications in the pediatric population.

## RESUMO

**Objetivo:**

Descrever o perfil das crianças atendidas na emergência de um hospital de referência pediátrica no sul do Brasil, vítimas de intoxicação exógena aguda entre 2016 e 2021, caracterizar os agentes tóxicos e descrever os fatores que se relacionam à internação hospitalar.

**Métodos:**

Estudo descritivo e analítico, com dados de prontuários de hospital pediátrico entre julho de 2016 e junho de 2021, por consulta às fichas de notificação compulsória. Foram avaliadas características das vítimas; do incidente; tipo de substância envolvida e classe; procedimentos realizados; necessidade de internação hospitalar, desfecho e necessidade de UTI. Calcularam-se frequências absolutas e relativas para as variáveis categóricas e medidas de tendência central e dispersão para as numéricas. Regressão logística binária foi realizada para identificar as variáveis associadas à internação.

**Resultados:**

Houve 411 atendimentos, com idade média de 7,2±5,5 anos, predomínio do sexo feminino (59,9%). A maioria das intoxicações ocorreu em domicílio (82,1%) e por via oral (93,7%). Produtos químicos ou de limpeza foram majoritários nas crianças com até um ano de idade, sendo os medicamentos os principais agentes nas demais faixas etárias. Resultaram em internações 38,7% dos casos, sendo as variáveis associadas: quantidade de agentes envolvidos, tipo de substância envolvida, medicamento que atua no Sistema Nervoso Central, recorrência, motivação (acidental/intencional) e realização de exames simples.

**Conclusões:**

Mais ações preventivas são necessárias, como legislações e maior orientação aos pais sobre a forma de armazenamento de produtos em ambiente doméstico, de modo a reduzir o número de intoxicações exógenas na população pediátrica.

## INTRODUCTION

Poisoning is a set of harmful effects with clinical or laboratory manifestations resulting from organic imbalance produced by the interaction between the toxic agent and the biological system.^1,2^ Intoxication may be due to ingestion, inhalation, transcutaneous absorption or other forms of exposure,^3^ and may cause transient or permanent damage, and even death if the individual is not rescued in time.^4^

According to the World Health Organization (WHO), acute intoxications lead to over 40,000 deaths annually worldwide, with an incidence of 1.6 per 100,000 inhabitants.^5^ Among the ages of 15 and 19, intoxications represented the 13^th^ cause of death around the world in 2014.^5^ Poisoning resulted in a substantial number of hospital admissions, incurring in a cost of more than 120 thousand reais for the Unified Health System (SUS) in March 2020 throughout Brazil, considering all age groups.^6^

The domestic environment is the most frequent place where this injury occurs, as it gathers a variety of harmful agents such as plants, medications, pesticides, hygiene, and cleaning products, which pose a risk of intoxication when not stored or used in a safe way.^4,7-9^

Acute unintentional poisoning at home is amid the main cause of domestic accidents in the child and youth population, and occupy a prominent position among the causes of emergency service visits.^4,6^ Caregivers’ unawareness about the agents’ toxicity, inattention to risks and lack of supervision contribute to the occurrence of accidental poisoning in childhood.^9^

Examining and understanding the epidemiology of accidental poisoning is essential to facilitate the development of educational measures and the implementation of preventive strategies.

Thus, this study intends to describe the profile of children victims of acute exogenous intoxication treated at a reference pediatric emergency unit, as well as characterize the main agents and environments involved, and present the factors related to hospitalization.

## METHOD

Retrospective, observational study, with data collection from medical records of patients who were victims of acute intoxication treated at the emergency room of a pediatric hospital in Santa Catarina (SC), from July 1^st^, 2016, to June 30^th^, 2021, and whose compulsory notification forms were filled with “acute intoxication” and aged from 0 to 15 incomplete years. Exclusion criteria were attendances out of the age range, lack of information regarding the type of substance involved, and incidents not considered poisonings after checking medical records (for instance, venomous animals or medication side effects within therapeutic dosage).

Victim-related exposure variables were categorized into: age (in age groups: 30d–1y, 2y–5y, 6y–9y, 10y–11y, 12y-–14y), race (white and non-white) and city of residence (Florianopolis or other). Variables concerning the incident: route of exposure (oral and non-oral), time elapsed between intoxication and hospital care (more or less than 3 hours), place of occurrence (home, school, others), number of toxic agents involved, motivation (accidental or intentional), presence of recurrence, the person responsible for the administration (child or third party), type of product related to the intoxication (medicine, chemical or cleaning products, pesticides or agrochemicals, licit/illicit drugs, others), drug class in case of intoxication with medicines, and need for procedures (simples tests, such as blood samples, radiography or electrocardiogram; additional exams, such as computed tomography, lumbar puncture or digestive endoscopy; or specific therapy, such as gastric decontamination or antidote use). Children who required medical observation for more than 10 hours in the emergency care were hospitalized, following the protocol of the institution. Hospitalization was considered the outcome. The need for admission in the Intensive Care Unit (ICU) was also evaluated.

Data were analyzed using descriptive statistics in a simple frequency and proportion. Binary logistic regression was used, adopting chi-square or Fisher’s exact tests in the crude model (variables with p<0.20). For the adjusted analysis, the backward selection method was chosen. In the logistic regression analyses, the results were expressed as odds ratios (OR) and respective 95% confidence intervals (CI). Statistical analyzes were performed using the Statistical Package for the Social Sciences; version 22.0. For all analyses, p<0.05 was considered significant.

The study was approved by the Research Ethics Committee of the researchers’ institution, under No. 5.144.226/2021.

## RESULTS

There were 427 consultations, 16 of which were excluded (4 not found, 4 not considered poisonings and, in 8, the substance involved was not possible to distinguish), resulting in 411 eligible attendances for data collection. There was no record of fatal intoxication or involvement in children younger than 30 days of life.

The mean age was 7.2±5.5 years, with 224 consultations (54.5%) occurring in children younger than 6 years old. Age groups between 6 and 11 years were the least affected, with an increase in cases in children aged 12 years or more (35.7% of the cases).

The characteristics of gender and race distribution among age groups are listed in Table 1. Florianópolis was the municipality of residence of 222 (54.0%) registered cases, and 81.0% of the children were taken to care by one or both parents.

**Table 1.** Characteristics of victims of acute intoxication at Hospital Infantil Joana de Gusmão, Florianópolis, SC, 2016–2021, by age group (n=411).

Variables	n (%) or mean (SD)	Age group (years)					p-value
		30 days–1 n (%)	2–5 n (%)	6–9 n (%)	10–11 n (%)	12–14 n (%)	
Number		106 (25.8)	118 (28.7)	24 (5.8)	16 (3.9)	147 (35.7)	
Age	7.2 (5.57)						
Sex							
Female	246 (59.9)	60 (56.6)	52 (44.1)	9 (37.5)	6 (37.5)	119 (81.0)	<0.001*
Race							
White	377 (91.7)	101 (95.3)	107 (90.7)	19 (79.2)	16 (100.0)	134 (91.2)	0.101^†^

SD: standard deviation; *Chi-square test; ^†^Fisher’s exact test.

Regarding intoxication, most occurred at home (82.1%) and orally (93.7%). In almost 1/5 of the consultations (77), there was exposure to more than one agent, ranging from 2 to 8, with an average of 1.4 products in each consultation. Children aged 12 years or older ingested a greater number of agents when compared to age groups below 10 years (p<0.05). In relation to motivation, 69.8% of intoxication cases were accidental and 30.2%, intentional, considering all age groups. In 15.6% of the consultations there was a report of acute intoxication at least once previously (recurrence). The variables “place of occurrence,” “number of agents involved,” “motivation” and “recurrence” were statistically significant (p<0.05) in relation to the division by age groups (Table 2).

**Table 2.** Characteristics of incidents of acute intoxication at Hospital Infantil Joana de Gusmão, Florianópolis, SC, 2016–2021, by age group (n=411).

Variables	Total n (%)	Age group (years)					p-value
		30 days–1 n (%)	2–5 n (%)	6–9 n (%)	10 –11 n (%)	12–14 n (%)	
Number		106 (25.8)	118 (28.7)	24 (5.8)	16 (3.9)	147 (35.7)	
Place (n=385)*							
Own domicile	316 (82.1)	87 (87.0)	102 (88.7)	17 (81.0)	12 (85.7)	98 (72.6)	<0.001^†^
Third-part domicile	28 (7.3)	11 (11.0)	10 (8.7)	3 (14.3)	0	4 (3.0)	
School	9 (2.3)	0	0	0	1 (7.1)	8 (5.9)	
Others^‡^	32 (8.3)	2 (2.0)	3 (2.6)	1 (4.8)	1 (7.1)	25 (18.5)	
Route (n=410)*							
Oral	384 (93.7)	95 (90.5)	111 (94.1)	22 (91.7)	13 (81.3)	143 (97.3)	0.052^†^
N of agents^§^	1.4±0.96	1.0±0.0	1.1±0.24	1.2±0.83	1.5±0.89	1.9±1.37	<0.001^//^
Motivation							
Intentional	124 (30.2)	0	1 (0.8)	1 (4.2)	9 (56.3)	113 (76.9)	<0.001^†^
Recurrence							
Yes	64 (15.6)	2 (1.9)	4 (3.4)	3 (12.5)	4 (25.0)	51 (34.7)	<0.001^†^

* Data without information from all records; ^†^Fisher’s exact test; ^‡^Others: public square, street, beach; ^§^Data expressed in mean±standard deviation; ^//^Anova one-way test.

In 374 consultations (90.9%) the child picked up the toxic agent, even if inadvertently, and in the others (9.1%) the caregiver made a mistake in dosing, or changed packaging. In 59.1% of the cases, the time elapsed until arriving at the emergency department was more than three hours.

**Figure 1.**
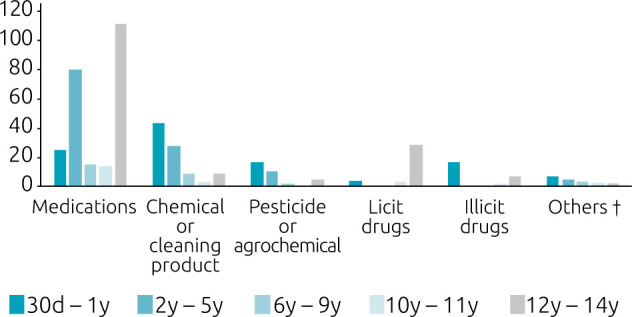
Distribution of the categories of substances involved in acute poisoning at Hospital Infantil Joana de Gusmão, Florianópolis, SC, 2016–2021, in absolute numbers (n=422)*

Regarding the substance involved, chemical or cleaning products were the most mentioned in children up to 1 year old; in the other age groups, the main agent was medication. The second position varied according to the age group: medicines in those up to 1 year of age, chemical or cleaning products in the age groups between 2 and 11 years and licit drugs in those aged 12 and over. In children up to 1 year of age, the third and fourth most common agents were pesticides or agrochemicals and illicit drugs (Figure 1).

*n>411 due to cases with exposure to more than one toxic agent; ^†^Others: gouache ink, product for veterinary use, toxic plant, helium gas used in balloons, hair gel; d: days; y: years.

Of the 32 cases of acute intoxication by licit drugs, alcohol was the most frequent (n=28), predominantly in the age group of 12 years or more. Among the 23 cases with illicit drugs, marijuana was the most listed (n=15), being more frequent in children under 1 year of age (53.4%), who ingested drug residues present on the floor. Rat poison was the most common among pesticides or agrochemicals (n=13) and, among chemicals or cleaning products, bleach (n=17) and caustic soda (n=15) had similar frequencies.

**Figure 2.**
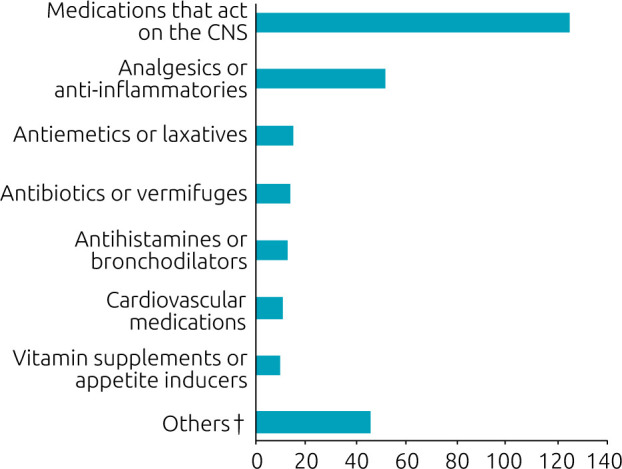
Distribution of medication classes involved in acute poisoning at Hospital Infantil Joana de Gusmão, Florianópolis, SC, in the period 2016–2021, in absolute numbers (n=286)*

When medicines were involved in intoxication (n=237), those who work on the Central Nervous System (CNS) were responsible for 125 intoxications, more than half (52.7%) of incidents with medicines, and almost 1/3 of the total number of poisonings included all categories of toxic agents. The second most frequent class was analgesics and anti-inflammatories, in 52 consultations (21.9%). The 10 drugs most involved in accidents were: clonazepam (n=30), paracetamol (n=22), dipyrone (n=15), amitriptyline (n=14), diazepam (n=14), fluoxetine (n=11), sertraline (n=11), naphazoline (n=10), levothyroxine (n=9) and risperidone (n=9) (Figure 2).

*n=286 due to cases with exposure to more than one medication; CNS: Central Nervous System; ^†^Others: thyroid pills, topical vasoconstrictors, muscle relaxants, eye drops, contraceptives, immunosuppressants, herbal medicines.

Considering the 411 consultations, 192 (46%) children were discharged without the need for any type of additional test, and the others required one or more of the options listed below: 202 children (49.1%) underwent simple blood or urine test, radiography, or electrocardiogram; 25 (6.1%) required more complex or invasive tests, such as computed tomography, upper digestive endoscopy or lumbar puncture; and 57 (13.8%) needed specific medication, gastric decontamination or antidote.

Hospitalization occurred in 159 cases, representing 38.7% of notifications. Of these, 7.5% (12 cases) were referred to the ICU. In the multivariate analysis, after adjustment (Table 3), hospitalization was related to the number of agents involved — the chance of hospitalization increased 1.81 times for each harmful substance ingested (95%CI 1.18–2.69); the type of product — chemical or cleaning products presented 2.21 times (95%CI 1.04–4.72) more chance when compared to those who ingested other substances; the medication class — the chance of those who act on the CNS was 2.39 times (95%CI 1.21–4.72) greater when compared to those who ingested other classes of medications; recurrence — 3.42 times (95%CI 1.48–7.90) more chance of hospitalization compared to those with no previous history; motivation — those who were intentionally intoxicated were 4.24 times (95%CI 2.01–8.91) more likely to be admitted to hospital when compared to patients with accidental intoxication; and undergoing simple tests — 7.62 more chance of hospitalization (95%CI 4,11–14,13) compared to those who did not need tests.

**Table 3.** Association between the characteristics of acute exogenous poisoning and hospitalization reported at Hospital Infantil Joana de Gusmão, Florianópolis, SC, 2016–2021 (n=411)

Variables	Categories	Outcome		Crude odds ratio (95%CI)	Adjusted odds ratio^†^ (95%CI)
		Discharge n (%)	Hospitalization n (%)		
Sex	Female	131 (52.0)	115 (72.3)	2.41 (1.58–3.70)	
	Male	121 (48.0)	44 (27.7)	1	
Route*	Oral	230 (91.6)	154 (96.9)	2.81 (1.04–7.62)	
	Non-oral	21 (8.4)	5 (3.1)	1	
Motivation	Accidental	226 (89.7)	61 (38.4)	1	1
	Intentional	26 (10.3)	98 (61.6)	13.97 (8.33–23.41)	4.24 (2.01–8.91)
Substances	Medication				
	Yes	111 (44.0)	126 (79.2)	4.85 (3.07–7.66)	
	No	141 (56.0)	33 (20.8)	1	
	Chemical or cleaning product				
	Yes	67 (26.6)	20 (12.6)	0.40 (0.23–0.69)	2.21 (1.04–4.72)
	No	185 (73.4)	139 (87.4)	1	1
	Pesticide or agrochemical				
	Yes	27 (10.7)	3 (1.9)	0.16 (0.05–0.54)	0.22 (0.04–1.14)
	No	225 (89.3)	156 (98.1)	1	1
	Licit drug				
	Yes	25 (9.9)	7 (4.4)	0.42 (0.18–0.99)	0.33 (0.10–1.09)
	No	227 (90.1)	152 (95.6)	1	1
Agents recurrence	Number (mean±SD)	1.09 (0.47)	1.81 (1.31)	3.48 (2.28–5.31)	1.78 (1.18–2.69)
	No	239 (94.8)	108 (67.9)	1	1
	Yes	13 (5.2)	51 (32.1)	8.68 (4.53–16.63)	3.42 (1.48–7.90)
Medication	Acting on the CNS				
	Yes	35 (13.9)	90 (56.6)	8.08 (5.03–13.00)	2.39 (1.21–4.72)
	No	217 (86.1)	69 (43.4)	1	1
Procedures	Simple tests				
	Yes	78 (31.0)	124 (78.0)	7.90 (4.99–12.53)	7.62 (4.11–14.13)
	No	174 (69.0)	35 (22.0)	1	1
	Additional exams				
	Yes	1 (0.4)	24 (15.1)	44.62 (5.97–333.46)	
	No	251 (99.6)	135 (84.9)	1	
	Specific therapy				
	Yes	25 (9.9)	32 (20.1)	2.29 (1.30–4.03)	1.46 (0.67–3.18)
	No	227 (90.1)	127 (79.9)	1	1

CI: confidence interval; SD: standard deviation; CNS: Central Nervous System; *Without information from all records; ^†^Analysis adjusted for: motivation, chemicals/cleaning substances, pesticides/agrochemicals and licit drugs, number of agents, recurrence, drugs acting on the CNS, simple tests and specific therapy; Hosmer and Lemeshow test: 0.633.

## DISCUSSION

Intoxication is one of the most common accidents in the youth population and occupies a prominent position among the causes of emergency service visits.^4,6^ Based on this study, 411 cases of acute poisoning were registered, an average of 8 to 9 cases per month, and 38.7% of victims required hospitalization.

Victims under the age of 6 predominated, followed by those aged 12 years or older, with fewer records in the age group between 6 and 11 years old, corroborating Vilaça et al., in which 72.6% of the victims of acute accidental poisoning were children from 0 to 4 years.^7^ For Nistor et al., 85.0% of intoxication cases were in children under 6 years of age.^10^ A Spanish study showed two incidence peaks, with 70.0% of intoxication cases occurring in children under 5 years of age, and 24.6% of cases in those over 12 years old.^8^ The fact that younger children are the most affected reflects the naivety inherent to their age, combined with the lack of discernment about the risks that toxic agents represent, pointing to the need for their strict surveillance.

Regarding gender, considering all age groups, females prevailed (59.9%). However, under 12 years old, there was a slight predominance of poisoning in males, while for those over 12 years old female victims prevailed.^7^ Notifications corresponding to white victims predominated, which was consistent with another study conducted in a mostly white population,^11^ a demographic characteristic also present in SC.^12^

Florianópolis was the most listed municipality of residence, corroborating other studies that indicate that the majority of those affected resided in the same municipality as the place of assistance.^3,7^ Despite the hospital being a reference in the state, a significant portion of the poisonings were not serious, and could be treated in their hometowns. Allied to this, the Center for Information and Toxicological Assistance of SC (CIATOX) has state coverage 24 hours a day, both for laypersons and professionals, contributing to adequate support for patients in their cities of origin, and reducing the unfavorable evolution of cases and the need for transfer to the reference center. In the present sample, similarly to another study,^4^ one or both parents were the main responsible for driving the minor to hospital care, which emphasizes the dependence that the pediatric population has on their caregivers.

As for the characteristics of the incident, the most frequent place of occurrence was the home (82.1%), a finding similar to studies in Spain (79.3%),^8^ Romania (95%),^10^ and in another Brazilian capital (90.0%).^7^ The most described exposure route, as in other studies,^7,10,11^ was oral (93.4%). These data reinforce the idea that the home, despite being considered a safe place, contains numerous potentially toxic substances, and that children need vigilance, in addition to the peculiarity of this phase of life: exploration of the environment, essential for neurocognitive development. In the first years of life, exploration of objects occurs mainly by putting them in the mouth, which favors a greater risk of accidental intoxication through this route in small children.^4^

In the present study, 81.3% of intoxications were caused by only one toxic agent, similarly to what was found by Vilaça et al.^7^ The ingestion of more than one harmful substance is significantly related to the risk of hospitalization, a fact probably explained by the interaction between the toxic agents in the organism, in a harmful and summative way.^2^

Regarding motivation in this sample, considering all age groups, most cases were accidental. However, it is noted that, in those over 12 years of age, this proportion is reversed, being mostly deliberate. Children aged 12 and over have a sense of danger and an understanding of the concept of “action and consequence.” Thus, adolescents are expected to have a lower risk of accidental intoxication than younger children,^2^ a fact described in other studies,^3,8^ but suicide attempts are more frequent.^3^

In addition to intentional poisoning being more frequent in those over 12 years of age, it predominates in females, tending to recurrence.^3,13^ Intentional injuries are the expression of a crisis process that develops gradually until it culminates in a suicide attempt.^13^

Early and adequate interventions, involving the individual and their set of relationships, are essential strategies for preventing this unwanted outcome.^14^

The authorship of the accidents, a data not found in other studies in the pediatric population, was related to the child themself (90.9%) — they caught the toxic agent, either intentionally or inadvertently. In the other cases, the author of the poisoning was not the child, emphasizing the possibility of the caregiver making dosage errors, swapping medications and agents, offering the child a known toxic substance, due to lack of attention. Even though the caregivers are responsible for only one tenth of the cases, the numbers support the guidance to parents/caregivers to check the packaging and prescribed dose before administering the medication, since it is still a considerable portion of the accidents that can be prevented by this action. Data from CIATOX^12^ point out that 1.4% of the intoxication cases occurred due to medication errors.

Regarding the time elapsed between intoxication and care at the emergency unit, like in another Brazilian study,^4^ a delay greater than 3 hours predominated, with no significant association with the outcome of hospitalization, a fact related to the possibility that these intoxications were not severe.

Medications lead the rankings of substances involved in intoxication,^1,11,15-18^ as observed in the present study, apart from children under 1 year of age, whose causal agents were chemicals or cleaning products. In the United States of America (USA), 50.7% of cases of acute poisoning in children under 5 years of age were caused by oral medications, corresponding to 43,733 children in one year, while cleaning products reflected 13.2%, and personal care products, 4.7%.^11^ In Romania, 67.0% of accidental poisonings in childhood are related to non-pharmacological products, mainly domestic substances, rodenticides and pesticides, a fact possibly related to the rural population of that country being proportionally larger than those in this sample.^10^ Data from the State of Santa Catarina indicate that chemical and household cleaning products occupy second and third place, varying according to the year and age group.^1,15-18^

Incidents with legal and illegal drugs, although amounting to a minority of the cases in the present study, are no less important. One study pointed to alcohol intake as being responsible for 49.9% of acute intoxications, with the youngest patient affected being only 8 years old.^3^ With regard to illicit drugs, marijuana is described as the main agent involved in intoxications.^19-21^ In a sample in France, 29 children were intoxicated with it (mean age of 16 months), the majority (87.0%) in a residential environment.^19^ These data demonstrate the importance of guiding the caregivers to refrain from using these substances at home, ideally practicing abstinence, but in case of use preferably doing it without the presence of the child and with extra care when it comes to disposal.

In relation to poisoning with medicines, anxiolytics, analgesics, anti-inflammatories and anticonvulsants lead the rankings.^1,7,10,11,20^ Despite the differences in classification, it is possible to notice that the agents involved are similar and frequently used by the population, emphasizing once again the fact that intoxications occur with agents that are more readily available or easily accessible at home.^7,8,10^

Regarding the need to collect specific tests or medication, in 219 of the 411 consultations, at least one type of test or procedure was performed. These may have caused, in addition to pain at the moment of collection, side effects of medications, risks inherent to the procedures and expenses generated to the health system,^6^ which could have been avoided, given that intoxication is a preventable problem.^9^

The hospitalization rate found (38.7%) was higher than in the USA (13.3%),^11^ Belo Horizonte (12.0%)^7^ and Cuiabá (24.4%),^4^ a situation perhaps explained by the institutional protocol of hospitalization for those patients who remained under observation in the emergency room for more than 10 hours, and also related to the high rate of intentional poisoning. Suspected or confirmed cases of self-inflicted violence must be notified,^22^ hospitalization being mandatory in emergency situations, notably in cases of imminent risk to life or physical integrity^14^ — such as intentional exogenous poisoning.

The factors related to hospitalization in cases of acute intoxication in this study were the number of products ingested; the substance involved; the class of medication that acts on the CNS; recurrence; intentionality (in cases where ingestion was deliberate); and the performance of simple tests, similarly to the findings by Vilaça et al.^7^ in terms of intoxication by more than one toxic agent, but differing in relation to the second risk factor for hospitalization, which was, for those authors, residing in a municipality other than the place of care.

A probable limitation to the study is the secondary source of data, which may have been minimized by the manual checking of the notification forms and confirmation of data in the victims’ hospital records.

Acute poisonings represent an important public health issue, both in the Brazilian national territory and abroad. Many of these toxic exposures could be avoided by simple and effective measures that are widespread worldwide, especially in the family environment: keeping cleaning products out of the reach of children and in their original packaging, maintaining medications stored in a safe place; having caregivers pay closer attention when children use medication, as they may confuse the dosage or take it intentionally; frequent surveillance by parents, health units, school and other environments where children usually go, in relation to their psychological state, aiming at early diagnose in cases of depression, avoiding intentional poisoning.^9,23^

The data from this study allow us to conclude that more preventive actions are needed, such as legislation and supervision of child-resistant packaging for medicines and chemical/cleaning products, and greater guidance to parents on how to store products in a domestic environment, in order to avoid the stressful, expensive, and life-threatening outcome: hospitalization.
